# Detection of Benign and Malignant Tumors in Skin Empowered with Transfer Learning

**DOI:** 10.1155/2022/4826892

**Published:** 2022-03-24

**Authors:** Taher M. Ghazal, Sajid Hussain, Muhammad Farhan Khan, Muhammad Adnan Khan, Raed A. T. Said, Munir Ahmad

**Affiliations:** ^1^Center for Cyber Security, Faculty of Information Science and Technology, Universiti Kebangsaan Malaysia (UKM), 43600 Bangi, Selangor, Malaysia; ^2^School of Information Technology, Skyline University College, University City Sharjah, 1797 Sharjah, UAE; ^3^Riphah School of Computing and Innovation, Faculty of Computing, Riphah International University, Lahore Campus, Lahore 54000, Pakistan; ^4^Department of Forensic Sciences, University of Health Sciences, Lahore 54000, Pakistan; ^5^Pattern Recognition and Machine Learning, Department of Software, Gachon University, Seongnam 13557, Republic of Korea; ^6^Faculty of Management, Canadian University, Dubai 117781, UAE; ^7^School of Computer Science, National College of Business Administration and Economics, Lahore 54000, Pakistan

## Abstract

Skin cancer is a major type of cancer with rapidly increasing victims all over the world. It is very much important to detect skin cancer in the early stages. Computer-developed diagnosis systems helped the physicians to diagnose disease, which allows appropriate treatment and increases the survival ratio of patients. In the proposed system, the classification problem of skin disease is tackled. An automated and reliable system for the classification of malignant and benign tumors is developed. In this system, a customized pretrained Deep Convolutional Neural Network (DCNN) is implemented. The pretrained AlexNet model is customized by replacing the last layers according to the proposed system problem. The softmax layer is modified according to binary classification detection. The proposed system model is well trained on malignant and benign tumors skin cancer dataset of 1920 images, where each class contains 960 images. After good training, the proposed system model is validated on 480 images, where the size of images of each class is 240. The proposed system model is analyzed using the following parameters: accuracy, sensitivity, specificity, Positive Predicted Values (PPV), Negative Predicted Value (NPV), False Positive Ratio (FPR), False Negative Ratio (FNR), Likelihood Ratio Positive (LRP), and Likelihood Ratio Negative (LRN). The accuracy achieved through the proposed system model is 87.1%, which is higher than traditional methods of classification.

## 1. Introduction

Cancer is the most commonly known, developing, and most dangerous disease all over the world [1]. Skin cancer is one of the types of cancer. According to the present measures of the World Health Organization (WHO), 2 to 3 million nonmelanoma and 132000 melanoma skin cancer cases turn out globally each year. Out of three diagnosed, one is skin cancer according to the reports of Skin Cancer Foundation Statistics (SCFS) [2]. Skin cancer emerges from the skin. It is an unusual growth of skin cells. These skin cells can invade the other body parts' cells. Maximum cases come out due to the Ultraviolet (UV) rays of the sun. But cases also come out on areas of body parts which are not ordinarily exposed to sunlight. Skin cancer is further divided into three main types: basal cells carcinoma (BCC), squamous cell carcinoma (SCC), and melanoma [3]. BCC is caused by damaged cells and causes changes in DNA, the basal cells of the outer layer of skin [4]. SCC of skin is caused by exposing skin to UV radiation for a longer duration. This radiation may be from sunlight or from lamps or any other such type of source [5]. Melanoma can spread from any part of our body. This disease attacks our normal skin and makes it cancerous. Melanoma mostly appears on the face or the trunk of affected men. In women, melanoma mostly appears on lower body parts like legs. Mostly, it spreads out on those parts of the body that are not directly exposed to sunlight [6]. In the detection of malignant tumors, many challenges have been faced. The factors such as images of different shapes and sizes, presence of noise in images, tumors irregular boundaries, and similarities with neighbor's tumors confused right identifications. For this purpose, image analysis methods were followed [7]. Computer-Aided Systems (CADs) identified border detection and extract required features. In CADs, different classification algorithms that automatically classified lesions into their relevant class are used [8]. One of them is AlexNet. It is a modest and strong CNN model consisting of convolutional and pooling layers and some fully connected layers [7]. In the proposed system, the problem is to classify benign and malignant tumors of the skin. Malignant tumors are severe types of tumors that grow and spread uncontrollably. These are called cancerous. While benign tumors remain there where they appear, they do not spread across other areas of the body. They are not much more problematic. The detection of skin cancer in starting days can be treated well and chances of recovery are high. Through novel techniques, this is possible [9]. The novel approaches of deep learning methods are mostly applied not only for skin cancer but also for other varieties of cancers like breast cancer, brain cancer, lungs cancer, and prostate cancer. In the literature, more algorithms for skin cancer images classification are proposed. But this is still a very challenging task. The problem must be addressed in ways as follows: skin cancer images boundaries might have full contour with maximum curves and small angles. It is still a question that how many images of skin cancer are needed to be trained and analyzed, partially answered [10], and still, it is an issue. In the proposed system model, a large dataset is used to handle this problem. Through this, we achieved maximum accuracy. It is relatively previous work better to deal with this problem. In this article, image base dataset of benign tumors and malignant tumors is used. A customized AlexNet model is applied. The AlexNet model is customized according to the existing problem for achieving high results. The customized existing AlexNet model consists of a total of 25 layers. There are 5 convolutional layers in this model. The initial layers are fixed and already trained. The last three layers are customized according to the proposed system output classes. It is a binary classification problem. So, the softmax layer is changed and set labeled according to output classes. The customized AlexNet layers are trained according to the dataset which is used in this proposed system. The input parameters for fully connected layers are the size of output classes which is of binary classification. Softmax layers applied softmax functions on providing input data. Fully connected layers are modified according to our classes' particular features. After that, these fully connected layers are able to train the model according to the classes' specific features.

The rest of the paper is set as follows. In Section 2, related work is described, Section 3 describes the material and methods which are used for this prediction, Section 4 comprises simulation and discussion of the result of the paper, and Section 5 is about the conclusion.

## 2. Literature Review

In the literature review, most of the representative approaches were used for skin cancer images classification. Diagnosis of any disease is very important for further proceeding and treatment. The same is in the case for skin disease; it is a challenging task for researchers to diagnose disease in its early stages. Different researchers applied different approaches to diagnose skin disease. These approaches include the following. Garg et al. proposed a system using Convolutional Neural Networks (CNNs) [11]. Masood and Al-Jumaily proposed a methodology that is tested on skin cancer datasets but it can be used and tested in any area where scarcity of labeled data is an issue. Their research demonstrates using data that is not a label for training the algorithm. For this purpose, they use a partially supervised method. The proposed research achieves 86.5% accuracy [12]. Mhaske and APhalke presented that Support Vector Machine (SVM) performs best rather than K-Mean's clustering and Neural Network (NN) for melanoma skin cancer and achieves 80% accuracy [13]. Fidan et al. did work on the ph2 dataset and presented that abnormal and melanoma skin cancer with NN and decision support system would help dermatologists in diagnosing skin lesions [14]. Amirreza and his fellow researchers developed a hybrid Deep Neural Network (DNN) for the classification of skin lesions. They used pretrained AlexNet, VGG16, and ResNet-18 for feature generators. After that, the SVM classifier was applied on 150 validation images, and for melanoma, 83.83% accuracy was achieved [15]. Rehman et al. presented work that consists of CNN for feature extraction, and after that, they used an ANN classifier for the detection of malignant lesions [16]. Monisha et al. presented ABCD dermoscopy for malignant recognition using backpropagation NN to rearrange harmful stage [17]. Albahar proposed a system using deep CNN applying a novel regularizer technique [18]. Jain presented work using Probabilistic Neural Network (PNN) classification for malignant lesions detection [19]. Maurya et al. presented that they used Grey Level Cooccurrence Matrices (GLCMs) for feature extraction, and after that, multiclass SVM applies and achieves 81.43% accuracy [20]. Pomponiu et al. presented DNN for feature extraction automatically and perform classification of skin lesions for malignancy on clinical dataset [21]. Esteva et al. presented using single CNNs train on 129,450 clinical images for versus benign and malignant melanomas versus [22]. In [23], a dataset with 19398 images was used for skin diseases classification, and the authors used an eight-layer CNN model on a dataset that has 900 images. Jianfeng He et al. constructed an 8-layer CNN model using a dataset containing 600 images for testing the model. Seifedine Kadry et al. proposed a system for the assessment of Skin Melanoma (SM) using a CNN-based approach. Using the VGG-SegNet scheme firstly, they extract the SM part From the Dermoscopy image. After that, the proposed technique was validated using the ISIC2016 database [24]. Attique et al. proposed a system in which they used a segmented RGB images dataset, which they later passed through the DenseNet model, extracting features. For this purpose, average pool and fully connected layers are applied. Later on, the combined result is forwarded to the feature selection block for downsampling using the proposed entropy controlled least square SVM. For next, they used three different datasets for validation and then measure the performance of RCNN [25]. Oluwakemi et al. proposed using the SqueezeNet deep learning model to improve the data augmentation model for effective detection of melanoma skin cancer [26]. Pham et al. [27] expose a comparative study technique through which they reveal that the color and shape of melanoma lesions are useful for classification with benign lesions. In their technique, they apply six classifiers along with seven feature extraction methods, performing data preprocessing by taking two datasets, and revealed that Random Forest is the best classifier with an accuracy of 81.46%. Pai and Giridaran [28] build a system using a VGG-16 customized CNN model, classifying various seven types of skin lesions. This system predicts the most probable types of skin lesions from given with 78% accuracy. Emrah and Zengin presented research [29] on the “HAM10000” dataset in which they use the K-Fold Cross Validation technique to distinguish seven different classes for training and testing purposes. After that, they applied VGGNET-16 architecture and obtain 85.62% accuracy.

## 3. Limitation of Related Work and Contribution

In [12], semilabelled data is used, the size of the dataset was less, only 1050 images were used, and an SVM classifier was applied which was unable to gain a maximum score. Handcrafted features were used in their proposed system too.

In [15], a less number of images were used in this system to validate the system, only 150 images. So, the malignant class achieved 83.83% accuracy, which is less as compared to the proposed system.

A fixed number of weights were used in [15], so maximum accuracy was not achieved.

In [30], a less number of images were used to train and after that to validate the system. So, this system without augmentation gained only 80% accuracy which is not up to the mark.

In the approach proposed in [20], a less images dataset was used, only 359 images are used in their proposed work, a multiclass SVM algorithm was applied, and the system gained only 81% accuracy which is minimum to the proposed approach.

Contrary to work done before, the proposed approach in this paper does not rely on handcrafted features. In the proposed approach, transfer learning intends to apply with Deep Convolutional Neural Network (DCNN) AlexNet pretrained network. Moreover, the proposed system network model is trained on 1920 images; each class contains 960 images. The size of the images was the same in both classes. Firstly, features are extracted through DCNN, and after that, a customized pretrained network is trained. After that, the proposed system network model is validated on 480 images. These are the specialties of the proposed system model. So, it performs better than earlier approaches.

## 4. Materials and Methods

In this section, materials for the paper and work done on the proposed system are briefly described.

### 4.1. Dataset

**Figure 1:**
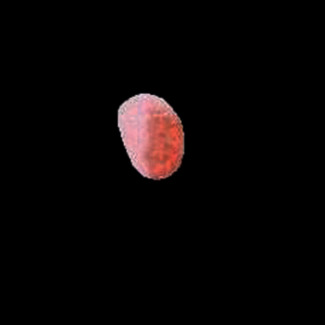
Class1.

**Figure 2:**
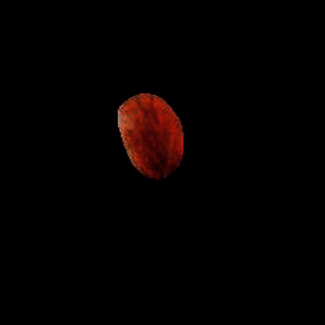
Class 2.

In the proposed system, a publicly available dataset is used taken from the Kaggle repository [29]. It is an images base dataset that consists of two different classes: class 1 is named malignantly segmented and the second one is named benignly segmented. Sample image of class 1 is given in Figure 1 and sample image of class 2 is given in Figure 2.

The original dataset consists of a total of 2637 images, the size of each class is imbalanced, and so 2400 images have been selected for the proposed system model where each class comprises 1200.

### 4.2. Experimental Setup

The proposed system model is developed by using pretrained AlexNet for the detection of malignant and benign tumors. MATLAB 2020a is being used for the classification and results. The proposed model of Detection of Malignant Benign Tumors (DMBTs) is further divided into two phases, training and validation. In the training phase, the model is trained on different epoch values of 10, 20, and 30; the learning rate of 0.001 is fixed in all epoch values. AlexNet has been trained on over a million images and classifies images into 1000 objects categories. In the proposed system, the pretrained AlexNet model is modified according to the problem taken in the proposed system. In the proposed model, the AlexNet model is trained on 1920 images that belong to two classes.

### 4.3. Model for Proposed System

**Figure 3:**
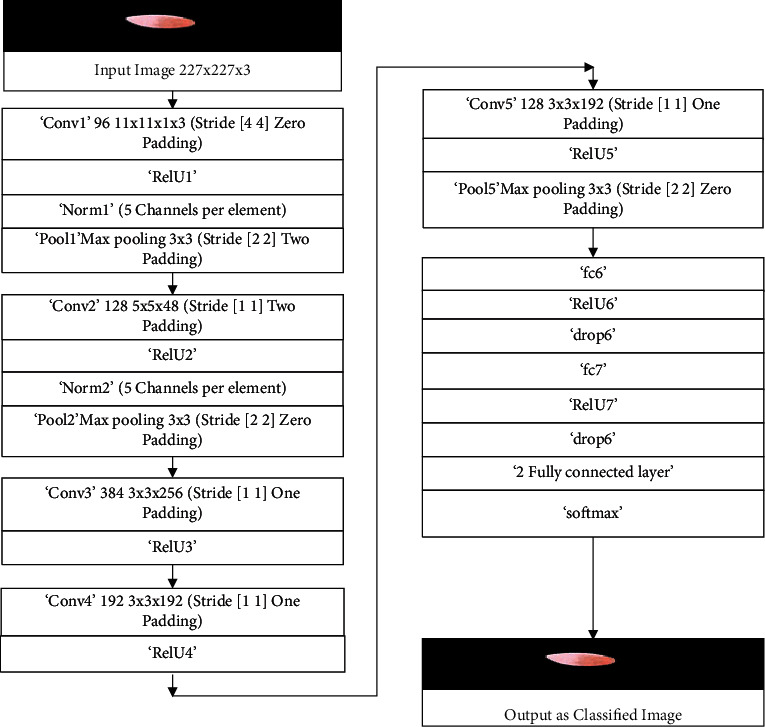
Proposed system model.

The graphical diagram of the proposed system model is given in Figure 3. The proposed model consists of a total of 25 layers. The first layer of the proposed model is the image input layer, and the dimension of the input image is 227 × 227 which is specified for the AlexNet model [15]. The RGB coloring scheme is used for input images. There are 5 convolutional layers in the proposed model.

RelU activation function is used for activation. Normalization and pooling are also done within different convolutions. The last three layers are modified according to the proposed system problem, respectively, with 2 fully connected layers against the weights given 2 × 4096 bias added 2 × 1 and tuned. The next is the softmax classification layer that classified input images according to option sets in the trained model. The last layer in the proposed system model is class output cross-entropy with classes benign and malignant.

### 4.4. Transfer Learning (Modified AlexNet)

One of the most famous techniques in the current era is deep learning, which is used in different fields of life such as the prediction of diseases, transportation, aeronautics, and agriculture. AlexNet is a pretrained convolutional network model. Transfer learning, a process of using a pretrained model, is commonly applied in deep learning applications [30]. Different deep learning pretrained models are used to tackle different types of real-world problems. In the proposed system model, the pretrained AlexNet model of deep learning is used for transfer learning intended for the detection and classification of malignant and benign tumors.

Table 1 represents the architecture of pretrained AlexNet which is composed of convolutional layers, pooling layers, and fully connected layers. AlexNet network model is a pretrained CNN network model and has a huge impact on the recently used application of deep learning. This CNN network is modified according to our problem requirement, and then, images were passed to our proposed modified AlexNet transfer learning network model. The last three layers are modified and customized according to our proposed system problem, and these layers are the output classification layer, fully connected layer, and softmax layer. The modified and customized network model is used for transfer learning.

### 4.5. Training and Validation Phase

In this phase, initially, pretrained AlexNet model is modified and then trained according to the proposed system problem. Firstly, we divide the dataset with a ratio of 80% and 20%. There are a total of 2400 images in the dataset which are used in the proposed model. After division, 1920 images are separated for training purposes, and the rest of 480 images would be used in the validation phase. Modified pretrained AlexNet model is trained on different epoch values of 10, 20, and 30, respectively. The learning rate of 0.001 is fixed in all epochs. After training, the phase model is validated on images that are separated already for validation purposes in the proposed system.

### 4.6. Data Acquisition Layer

In this layer, the data is acquired on which model has to be trained. There are a total of 2400 images in this dataset. It is a classification type dataset; classes' names are malignant and benign tumors. The model has to classify the input images into one of the classes: malignant or benign.

### 4.7. Data Preprocessing Layer

For further processing, first, we need to process data in such a form that will be more effective for the model. The original dataset is not in such a form that the model will be trained on it directly. Images are not according to the AlexNet requirements. AlexNet model only can be trained on data having a size of 227 × 227 and a coloring scheme of RGB. It is all done using Image Batch Processor.

### 4.8. Application Layer

Till now, the data acquisition process is completed and also data is set according to the requirements of the AlexNet model. The AlexNet model is trained on the training dataset which is used in this proposed system. And the results are computed according to the required parameters. The model is trained on different epoch values of 10, 20, and 30, respectively, in the proposed system.

### 4.9. Performance Evaluation Layer

The pretrained AlexNet model is trained in the training phase, and performance is evaluated in the validation phase. The performance of the model is checked through performance metrics. The results produced by the proposed pretrained AlexNet model are examined by using different evaluation metrics. The 1920 images with a ratio of 80% are taken for training purposes, and the rest of 480 images with a ratio of 20% are taken for system validation. Accuracy, Miss Rate (MR), sensitivity, specificity, Positive Predictive Value (PPV), False Predictive Value (FPV), False Negative Rate (FNR), False Positive Rate (FPR), Likelihood Ratio Positive (LRP), and Likelihood Ratio Negative (LRN) parameters are used to evaluate the performance of the proposed model. These evaluation metrics act as a tool to evaluate classification models and are used in measuring the performance of the predictive model.

## 5. Results and Discussion

The developed proposed system model uses a pretrained AlexNet model for the detection and classification of malignant and benign tumors. Some changes are made in this pretrained AlexNet model according to the proposed system problem. And further, this proposed system model is divided into two layers: training and testing. The proposed system model is trained before and after that validate on separated testing data. As mentioned earlier, 80% of the dataset is used for training purposes and the rest of the 20% is used to validate the proposed model. The produced results of the proposed model are evaluated using performance evaluation metrics. For performance measurement, performance parameters are used to measure the performance of the proposed system model. The following are performance measuring metrics through which performance is measured: Accuracy, Miss Rate (MR), sensitivity, specificity, Positive Predictive Value (PPV), False Predictive Value (FPV), False Negative Rate (FNR), False Positive Rate (FPR), Likelihood Ratio Positive (LRP), and Likelihood Ratio Negative (LRN). The following metrics are a good way to use to judge the performance of the proposed system which are as follows:

(1) $$\begin{array}{c} \text{accuracy}=\frac{TP+TN}{\text{total number of instancees}}\times 100\%, \end{array}$$

(2) $$\begin{array}{c} \text{miss rate}\left(\text{MR}\right)=1-\text{accuracy}\times 100\%, \end{array}$$

(3) $$\begin{array}{c} \text{sensitivity }=\frac{TP}{TP+FP}\times 100\%, \end{array}$$

(4) $$\begin{array}{c} \text{specificity }=\frac{TN}{TN+FN}\times 100\%, \end{array}$$

(5) $$\begin{array}{c} \text{positive predictive values}=\frac{TP}{TP+FN}\times 100\%, \end{array}$$

(6) $$\begin{array}{c} \text{false predictive values}=\frac{TN}{FP+FN}\times 100\%, \end{array}$$

(7) $$\begin{array}{c} \text{false positive rate }\left(\text{FPR}\right)=1-\text{specificity,} \end{array}$$

(8) $$\begin{array}{c} \text{false negative rate }\left(\text{FNR}\right)=1-\text{sensitivity,} \end{array}$$

(9) $$\begin{array}{c} \text{likelihood ratio positive}=\frac{\text{sensitivity}}{\text{false positive rate}}\times 100\%, \end{array}$$

(10) $$\begin{array}{c} \text{likelihood ratio negative}=\frac{\text{false negative rate}}{\text{specificity}}\times 100\%. \end{array}$$

The proposed system model is applied in the validation phase and it classifies malignant and benign tumors into one of the classes.

**Figure 4:**
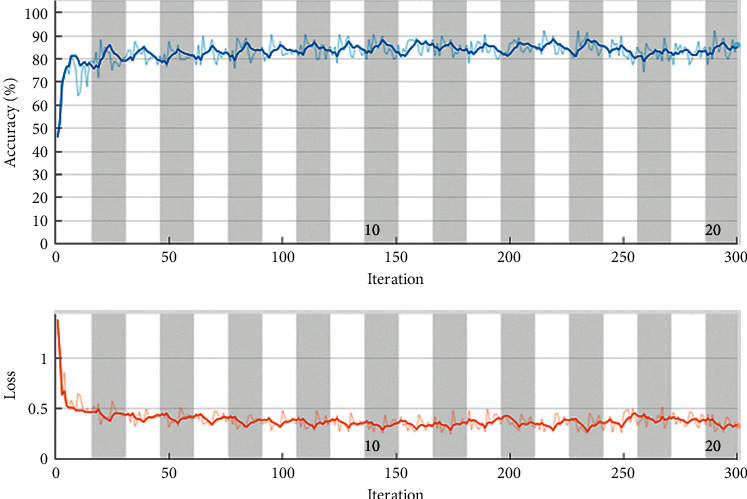
Training progress graph.

Table 2 represents simulation parameter values. The data is trained on multiple epochs like 10, 20, and 30, and maximum accuracy is gained through the proposed network model on 20 epochs. The training graph on epochs 20 where the system gains high accuracy is shown in Figure 2. Maximum accuracy is verified in the validation phase of all epochs, where the proposed model is validated on testing data. In Table 2, results are shown of all epochs. When the model is trained on an epoch value of 10, then it generates an overall accuracy of 82.1% and one of 82.4% from benign class while forming a malignant class of 81.8%. On an epoch value of 30, the model is trained and validated. In this training, the proposed model achieved an overall 83.8% accuracy. On 30 epochs, training and validation benign class achieves 83.2% accuracy while malignant class achieves 84.3% accuracy. On 20 epochs, the proposed model is trained and validated. In this validation, the proposed model achieves the highest accuracy of 87.1%. So, 20 epochs is selected for the proposed model. Here, the accuracy achieved through benign class is 93.2% while malignant class is not performing well, and 82.5% accuracy is achieved through the malignant class. For training, the proposed network model RelU activation function was used. The learning rate was fixed in all epochs which was 0.001 constant and the number of iterations was 300. Iterations per epoch were 15. Single CPU was used as a hardware resource. Time taken for this training was 49 minutes and 11 seconds as shown in Figure 4.

Table 2 represents the comparison of training and after that validation score on different epoch values, respectively, of 10, 20, and 30. Table 2 represents the score of different epoch values where the proposed network model achieved the highest score of 87.1% on an epoch value of 20.

**Figure 5:**
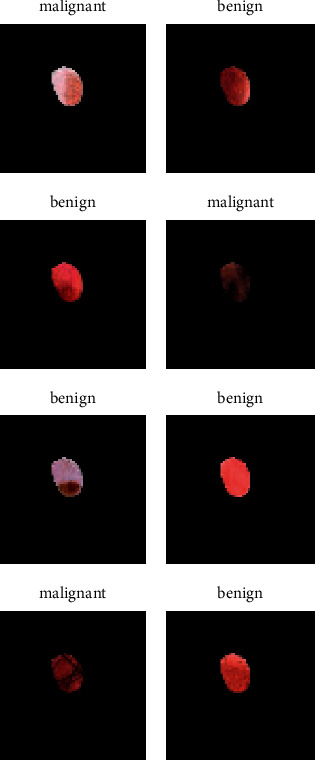
Classification images through the proposed system.

Figure 5 shows the labeled images of malignant and benign classes through the proposed system model. Total 8 images are given as input to model 4 from each class: proposed system model 5 of it in benign class while 3 in malignant class.

Table 3 expresses the proposed system confusion matrix during the validation phase. Before this, the proposed system model is trained on 1920 images, where each class consists of 960 images. After that, the proposed system model is validated; during validation, 480 images were used, where each class consists of 240 images. For this purpose, a value of 20 epochs was set. From benign class, the proposed system model classifies 192 images as correct while 48 images are classified as incorrect. In the malignant class, the proposed system model classifies 226 images as correct while 14 images as incorrect.

Table 4 represents the statistical performance measuring parameters like Accuracy, Miss Rate (MR), sensitivity, specificity, Positive Predictive Value (PPV), False Predictive Value (FPV), False Negative Rate (FNR), False Positive Rate (FPR), Likelihood Ratio Positive (LRP), and Likelihood Ratio Negative (LRN). The proposed system model achieved an accuracy of 87.1% accuracy in the validation phase on 20 epochs. The values above performance measuring parameters are given in the table. These values are achieved through formulas given in equations (1)–(10).

Multiple approaches have been used in the past for the detection of skin cancer, but transfer learning is the novel approach to detect and differentiate malignant and benign tumors. The proposed methodology achieved a high accuracy score in the detection of skin cancer disease. Thus, the proposed methodology helps medical consultants to identify disease and treatment for restraining the spread of disease.

Table 5 shows the proposed system model with previously published approaches. It is observed that the proposed model gives 87.1% accuracy which is higher than the approaches published earlier.

The proposed system model DCNN, transfer learning intend with customized pretrained AlexNet model, achieved higher accuracy than existing published approaches.

## 6. Conclusions

The earlier work done for skin cancer disease detection was not accurate enough to classify tumors whether they belong to the benign or malignant family. An automated framework is required that can classify tumors as benign or malignant. The proposed system, based on the transfer learning classification model, is able to handle this problem. It detects and classifies the family of tumors accurately. The proposed system model used pretrained AlexNet, retrained CNN. The customized pretrained AlexNet model was validated on the validation dataset and achieved 87.1% accuracy on 20 epochs. The proposed system model does not require handcrafted features. It is very fast and easily manageable for large datasets too. In future work, well-known datasets of skin lesions like Ph2, MED-NODE, DermIS & DermQuest, ISIC 2017, ISIC 2018, ISIC 2019, and ISIC 2020 will be used in different architecture. The AlexNet model can be made more efficient and accurate by fine-tuning all the convolutional layers and improving malignant class accuracy. So, net accuracy can be increased. The rest of the pretrained network can also be explored.

## Figures and Tables

**Table 1:** Architecture of AlexNet.

Layers	Conv1	Pool1	Conv2	Pool2	Con3	Conv4	Conv5	Pool5	FC6	FC7	FC
Kernel	11 × 11 × 3	3 × 3	5 × 5 × 48	3 × 3	3 × 3 × 256	3 × 3 × 192	3 × 3 × 192	3 × 3	—	—	—
Stride	[4 4]	[2 2]	[1.1]	[2.2]	[1.1]	[1.1]	[1.1]	[2.2]	—	—	—
Channels	96	96	256	256	384	384	256	256	4096	4096	4096

**Table 2:** Comparison of different epoch scores.

No. of epochs	Learning rate	No. of layers	Size of input images	Pooling method	Accuracy (%)
10	0.001	25	227 × 227 × 3	Max	82.1
20	0.001	25	227 × 227 × 3	Max	87.1
30	0.001	25	227 × 227 × 3	Max	83.2

**Table 3:** Confusion matrix for the proposed system model.

	Predicted class (benign)	Predicted class (malignant)
Input class (benign)	TP = 192	FN = 14
Input class (malignant)	FP = 48	TN = 226

TP represents the True Positive prediction, TN represents True Negative prediction, and FN shows False Negative prediction while FP indicates False Negative prediction.

**Table 4:** Performance evaluation table of the proposed model on 20 epochs in the validation phase.

Accuracy (%)	MR (%)	Sensitivity (%)	Specificity (%)	PPV (%)	NPV (%)	FPR (%)	FNR (%)	LRP	LRN
87.1	12.9	80.0%	94.2	93.2	82.5	.06	.02	14	2.13

**Table 5:** Comparison analysis of the proposed system with existing systems.

Study	Method	Year of proposed	Accuracy (%)
[12]	SVM	2017	86.5
[15]	CNN	2019	83.83
[30]	CNN	2019	80
[20]	SVM	2014	81
[27]	Random forest	2019	81.46
[28]	CNN	2019	78
[29]	CNN	2019	85.62
Proposed	DCNN, transfer learning intend with pretrained AlexNet	2022	87.1

## Data Availability

The data used in this paper can be requested from the corresponding author upon request.
